# Myocarditis and sports in the young: data from a nationwide registry on myocarditis—“MYKKE-Sport”

**DOI:** 10.3389/fspor.2023.1197640

**Published:** 2023-06-26

**Authors:** Isabelle Schöffl, Sophia Holler, Sven Dittrich, Thomas Pickardt, Bernd Opgen-Rhein, Martin Boehne, Bardo Wannenmacher, Katja Reineke, Gesa Wiegand, Tobias Hecht, Michael Kaestner, Daniel Messroghli, Stephan Schubert, Franziska Seidel, Annika Weigelt

**Affiliations:** ^1^ Department of Pediatric Cardiology, Friedrich-Alexander-Universität, Erlangen-Nürnberg, Germany; ^2^ School of Clinical and Applied Sciences, Leeds Beckett University, Leeds, Great Britain; ^3^ Competence Network for Congenital Heart Defects, Berlin, Germany; ^4^ Department of Pediatric Cardiology, Charité-Universitätsmedizin Berlin, Berlin, Germany; ^5^ Department of Paediatric Cardiology and Intensive Care Medicine, Hannover Medical School, Hannover, Germany; ^6^ Clinic for Paediatric Cardiology, Heart Centre, University of Leipzig, Leipzig, Germany; ^7^ Department for Paediatric Cardiology, University Heart Center Freiburg, Freiburg, Germany; ^8^ Department for Paediatric Cardiology, University Hospital Tübingen, Tübingen, Germany; ^9^ Heart- and Diabetes Center North Rhine-Westphalia, Center of Pediatric Cardiology and Congenital Heart Disease, Ruhr University Bochum, Bad Oeynhausen, Germany; ^10^ Pediatric Cardiology, University Hospital Ulm, Ulm, Germany; ^11^ Department of Cardiology, German Heart Center Berlin, Berlin, Germany; ^12^ Department of Cardiology, Charité-Universitätsmedizin, Berlin, Germany; ^13^ German Centre for Cardiovascular Research, Berlin, Germany; ^14^ Department of Congenital Heart Disease and Pediatric Cardiology, German Heart Center Berlin, Berlin, Germany; ^15^ Institute for Imaging Science and Computational Modelling in Cardiovascular Medicine, Charité-Universitätsmedizin Berlin, Berlin, Germany; ^16^ Experimental and Clinical Research Center, A Cooperation Between the Max-Delbrück-Center for Molecular Medicine in the Helmholtz Association and the Charité-Universitätsmedizin Berlin, Berlin, Germany

**Keywords:** heart muscle infection, return to sports, cardiomyopathy, sudden cardiac death, prevention

## Abstract

**Background:**

Myocarditis represents one of the most common causes of Sudden Cardiac Death in children. Myocardial involvement during a viral infection is believed to be higher as a consequence of intensive exertion. Recommendations for return to sports are based on cohort and case studies only. This study aims to investigate the relationship between physical activity and myocarditis in the young.

**Patient:**

Every patient in the MYKKE registry fulfilling criteria for suspicion of myocarditis was sent a questionnaire regarding the physical activity before, during and after the onset of myocarditis.

**Method:**

This study is a subproject within the MYKKE registry, a multicenter registry for children and adolescents with suspected myocarditis. The observation period for this analysis was 93 months (September 2013–June 2021). Anamnestic, cardiac magnetic resonance images, echocardiography, biopsy and laboratory records from every patient were retrieved from the MYKKE registry database.

**Results:**

58 patients (mean age 14.6 years) were enrolled from 10 centers. Most patients participated in curricular physical activity and 36% in competitive sports before the onset of myocarditis. There was no significant difference of heart function at admission between the physically active and inactive subjects (ejection fraction of 51.8 ± 8.6% for the active group vs. 54.4 ± 7.7% for the inactive group). The recommendations regarding the return to sports varied widely and followed current guidelines in 45%. Most patients did not receive an exercise test before returning to sports.

**Conclusion:**

Sports before the onset of myocarditis was not associated with a more severe outcome. There is still a discrepancy between current literature and actual recommendations given by health care providers. The fact that most participants did not receive an exercise test before being cleared for sports represents a serious omission.

## 1. Introduction

Myocarditis, defined as an “inflammatory disease of the heart muscle” is considered one of the most common acquired causes of arrhythmias, myocardial dysfunction, heart failure, and sudden cardiac death (SCD) in young individuals (1, 2).

### 1.1. Symptoms and diagnosis

Especially in the general population the exact incidence of myocarditis is unclear because of undiagnosed and asymptomatic cases (3). In athletes the incidence rate varies depending on the investigated cohorts but accounts for up to 8% of deaths (1, 4, 5). Myocarditis has been identified as the most common structural cause of Sudden Cardiac Death (SCD) in children in Denmark (6). A study using a nationwide registry on sports related SCD's (SRSD) in Germany also found myocarditis to prevail as cause in young athletes (<35 years of age) (7). Most cases of SRSD due to myocarditis were related to non-elite competitive or recreational activity (6, 7, 8). Almost all cases were preceded by upper respiratory tract infections (7). As a consequence of intensive exertion during physical activity (PA), the amount of myocardial involvement during viral infections has been shown to be higher in animal models and even a short break (8 days) from physical activity led to a significant decrease in mortality due to myocarditis (9). These findings stress the importance of clarifying the relationships between myocarditis and return to sports in the pediatric community.

There are recommendations for return to sport after myocarditis (10). These are based on case series and low-quality cohort and case studies. After suspected or diagnosed myocarditis athletes are recommended to stop training and competing for 3–6 months depending on the extent of reduction in ejection fraction and the myocardial damage seen in magnetic resonance imaging (MRI) (10). Most of the time, conservative approaches with long periods of restraint are suggested, with severe consequences for the children (11). Those, who participate in competitive sports face training losses and in some cases future careers may be at stake. However, in a time in which most children spend too much time with sedentary behavior (12), the decrease in cardiopulmonary fitness due to prolonged restrictions of physical activity with myocarditis could have detrimental effects for the affected children (13).

Another aspect is the fact that long periods of rest are not adhered to by the patients (11). Clear instructions based on evidence in combination with regular follow-ups for insuring compliance are therefore essential in the treatment of myocarditis in young patients and athletes.

This study aims to investigate the relationship between physical activity and the occurrence of myocarditis as well as the compliance to the provided recommendations.

## 2. Material and methods

This study is a subproject of the MYKKE registry, which is a multicenter registry for children and adolescents with suspected myocarditis (14). Patients with suspected myocarditis have been included since September 2013. The observation period for this analysis was 93 months (September 2013 to June 2021). Patients from the MYKKE registry meeting the following criteria were enrolled (15):

- Age ≥5 years and <18 years at first admission and
- Biopsy proven myocarditis or
- Myocarditis diagnosed in MRI [positive late gadolinium enhancement (LGE) and/or edema] or
- Electrocardiographic (ECG) abnormalities like ST-elevation and T-inversion or
- Elevated cardiac biomarkers (Troponin I and/or N-terminal pro brain natriuretic peptide (NT-proBNP).

We did not include subjects at a lower age as it is very difficult to assess physical activity in the very young.

The study was approved by the ethical committee of the Charité Universitätsmedizin Berlin, Germany and the Friedrich-Alexander-Universität Erlangen-Nürnberg, Germany.

Every patient from the registry fulfilling the mentioned criteria was then sent a questionnaire specifying the physical activity around the occurrence of the myocarditis as well as after 6 and 12 months.

The questionnaire was divided in several sections:

(1) 6 months prior to the onset of myocarditis the participation in curricular physical activities (PA) and the amount of extracurricular PA in type of sport and hours per week was established. It was also investigated whether the sport was performed as a competitive sport and how the children got to school (by bus/car, by bike or on foot)
(2) 2 weeks prior to the onset of myocarditis it was established whether the participant had practiced their sport as before, less or more, whether there were signs of infection and if so what kind of the infection (fever, sore throat, runny nose, diarrhea, other). Was a rest period undertaken.
(3) After treatment for myocarditis was a rest period recommended and if so for how long. What diagnostics were undertaken before return to sports (echocardiography, MRI, stress test, Holter ECG) and what recommendations were given for the return to sports (moderate intensity, individualized training programme, competitive sport) or were special sports prohibited (risk sports, contact sports, competitive sports). How long was the control interval?
(4) The compliance was established by asking whether the recommendations had been adhered to and if not with regards to competitive sports, risk sports, contact sports or intensity. How long was the true resting period before returning to sports and at what intensity.
(5) Symptoms after returning to sports were classified as: dyspnea, chest pain or rhythm disorders and subdivided into occurrence during PA or at rest.
(6) The occurrence of a recurrence as well as whether it had occurred during PA was recorded in a binary fashion.
(7) The question from 1) were then asked again after 6 and after 12 months.

The data from the MYKKE registry included anthropometric data (age, height, weight), data from MRI (LGE occurrence, EF), echocardiography, ECG (rhythm disorder, repolarization abnormalities), the laboratory (CRP, blood count, Troponin, NT/pro-BNP, virology), exercise test (VO_2_peak, patient characteristics (age, height, weight at the moment of myocarditis),.

### 2.1. Data analysis

Statistical analysis was performed using SPSS 24.0® (SPSS Inc., Chicago, IL). All measured values are reported as means and standard deviations or as median and interquartile range (P25 and P75) for non-Gaussian distributed data. The Kologomorov-Smirnov test was used to check for normal distribution. Homogeneity of variance was investigated using Levine's F-test. Parametric, normally distributed variables were investigated using Student *t*-test, non-parametric variables were investigated using *χ*^2^-test. A *p*-value of <0.05 was considered statistically significant.

## 3. Results

### 3.1. Demographics

An overall of 220 patients were contacted for completing the questionnaire. In 58 (26.4%) cases we were able to retrieve completed questionnaires. The mean age at the onset of myocarditis was 14.6 (±3.13) years of age (minimum 5.7 years, maximum 18 years). Most of the subjects were between 13 and 17 years of age (70.7%). There were more male subjects (65.5%) than females (34.5%). All the demographic data is represented in Table 1.

**Table 1:** Demographics of the participants with values given as absolute numbers with percentage of the total number in parentheses (%), or as means ± standard deviation.

	All Participants	Competition sports	No competition sports
	*N* = 58	*N* = 21	*N* = 32
Gender			
Male	38 (65.5)	15 (8.7)	19 (11)
Female	20 (34.5)	6 (3.5)	13 (7.5)
Characteristics			
Age (years)	14.60 ± 3.13	14.5 ± 2.8	14.8 ± 3.2
Height (cm)	163.7 ± 27.2	161.7 ± 29.0	165.2 ± 27.1
Weight (kg)	68.2 ± 29.5	65.2 ± 31.0	72.1 ± 29.6
Symptoms			
NYHA I	34 (58.6)	12 (7)	19 (11)
NYHA II	6 (10.3)	2 (1.2)	4 (2.3)
NYHA III	2 (2.3)	2 (1.2)	0
NYHA IV	7 (12.1)	1 (0.6)	5 (2.9)
NYHA n.a.	9 (15.5)	4 (2.3)	4 (2.3)
ECG			
ST-changes	19 (32.8)	9 (5.2)	8 (4.6)
T-inversion	19 (32.8)	8 (4.6)	9 (5.2)
AV block	36 (62.1)	15 (8.7)	17 (9.9)
Laboratory			
Leucocytes (Tsd/µl)	9.6 ± 5.0	12.6 ± 7.3	8.3 ± 2.8
CRP (mg/L)	15.6 ± 44.5	30.5 ± 75.1	10.2 ± 17.8
NT-proBNP (pg/ml)	2,491.0 ± 6,062.9	1,427.5 ± 2,079.8	3,131.9 ± 7,589.8
Troponin elevated	2,013.1 ± 3,851.5	1,611.0 ± 2,145.1	2,429.2 ± 4,775.8
Echocardiography			
LVEF (%)	56.3 ± 13.4	59.1 ± 8.5	55.8 ± 16.2
MRI	*N* = 48	*N* = 20	*N* = 23
LVEF (%)	54.6 ± 11.3	53.3 ± 7.1	54.9 ± 14.6
Oedema	15 (25.9)	6 (3.5)	8 (4.6)
LGE positive	31 (53.4)	14 (8.1)	14 (8.1)
Cardiopulmonary Exercise tests	*N* = 14 (24.1)	5 (8.6)	7 (12.1)
Devices			
Pacemaker	0 (0)	0 (0)	0 (0)
Cardiac catheter	28 (48.3)	11 (6.4)	14 (8.1)
VAD	5 (2.9)	0	4 (2.3)
ECMO	2 (1.2)	0	2 (1.2)
Complications			
Decompensation	7 (4.1)	1 (0.6)	5 (2.9)
Resuscitation	3 (1.7)	0 (0)	3 (1.7)
Death	0 (0)	0 (0)	0 (0)

MRI, cardiovascular magnetic resonance imaging; CRP, C-reactive protein; ECG, Electrocardiogram; ECMO, extracorporal membrane oxygenation; LGE, late gadolinium enhancement; LVEDVi, indexed left ventricular enddiastolic volume; LVEF, left ventricular ejection fraction; n.a., not applicable; NT-proBNP, N-terminal pro brain natriuretic peptide; NYHA, New York Heart Association; VAD, ventricular assist device.

### 3.2. Before the onset of myocarditis

**Figure 1:**
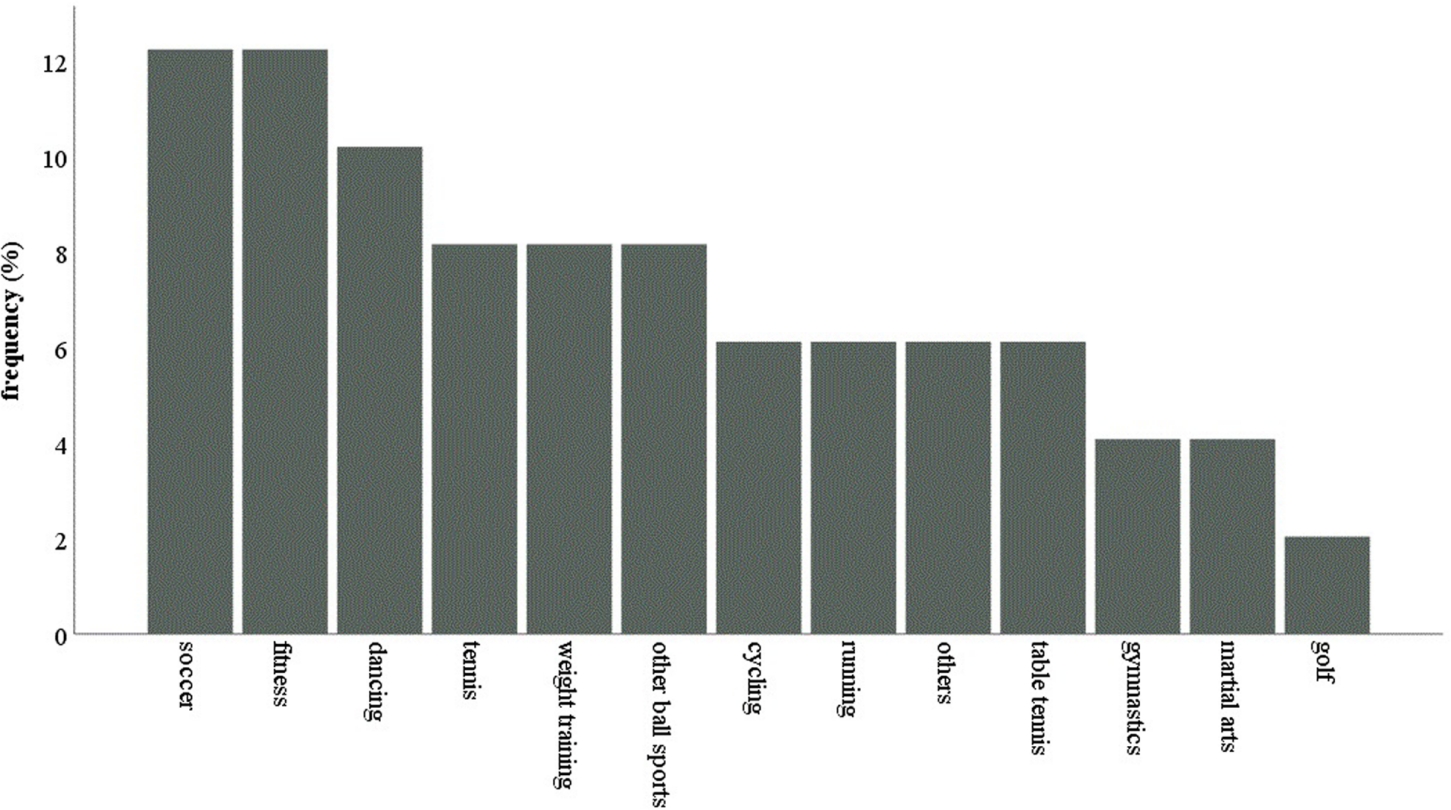
The different types of extracurricular physical activities performed by the subjects before the onset of myocarditis (in %).

Most of the subjects had participated regularly in school or kindergarten related physical activity (84.5%) 6 months prior to the onset of myocarditis. There was no significant difference between the subjects who had participated in curricular physical activity (PA) and those who had not with regards to the diagnostic parameters (left ventricular ejection fraction (LVEF) in echocardiography, LVEF in MRI, C-reactive protein (CRP), leucocyte count, NT-proBNP, Troponin, or the numbers of participants with ECG abnormalities (ST-changes, T-inversion or AV-Block) at onset of myocarditis. An overall of 21 subjects (36.2%) participated in competition sport. The subjects who competed in sports did not differ significantly from those who didn't with respect to the diagnostic parameters defined above (see Table 1). There were actually more left ventricular assist devices (LVAD) (4 vs.0) and extracorporeal membrane oxygenation (ECMO) patients (2 vs. 0) in the group of myocarditis patients who did not participate in competition sports. Figure 1 represents the different types of extracurricular physical activities performed by the subjects.

### 3.3. Around the onset of myocarditis

An overall of 35 (60.3%) subjects performed their sport as before 2 weeks before the onset of myocarditis, while 21 (37.9%) subjects had reduced their amount of PA and 2 patients (3.4%) had increased their physical activity compared to 6 months before. The two patients who were doing more sports 2 weeks prior to the onset of myocarditis did not experience more severe symptoms or showed signs of a more serious course of disease. They also did not experience symptoms of an infectious disease and thus did not feel the need to reduce their physical activity.

**Figure 2:**
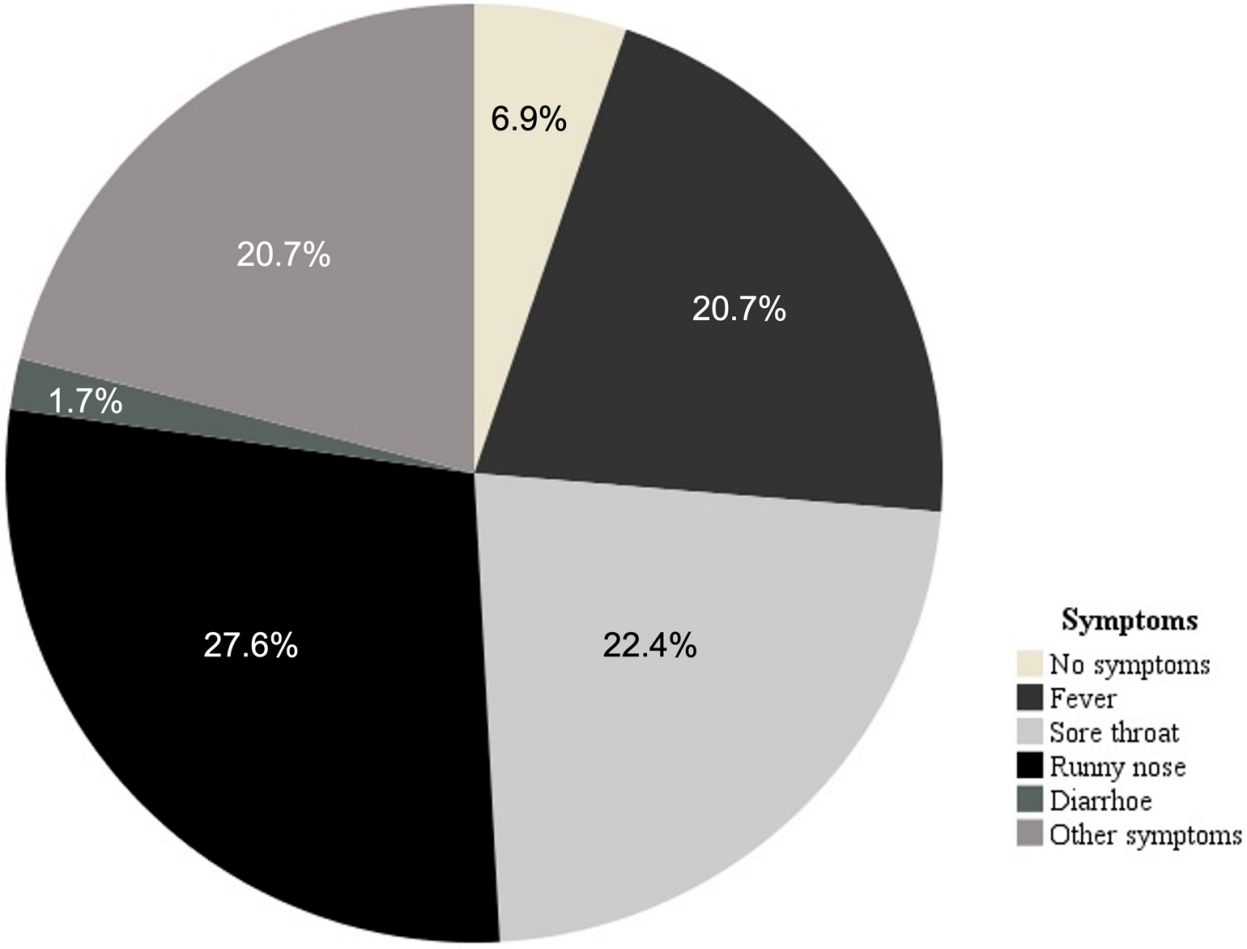
The frequency of symptoms reported by the subjects 2 weeks prior to the onset of myocarditis. Where subjects reported of more than one symptom, the more severe was listed (fever > sore throat or diarrhea > runny nose > unspecific symtpoms).

Symptoms of an infectious disease were reported by 54 (60.3%) participants. The other 4 participants (6.9%) reported no symptoms of an infectious disease. The frequency of the symptoms is depicted in Figure 2. Where subjects reported of more than one symptom, the more severe was listed (fever > sore throat or diarrhea > runny nose > unspecific symptoms). The severity of symptoms was ranked according to the guidelines referred to as the “neck-check”, in which localized symptoms of infectious disease are classified as above the neck and below the neck and recommendations for return to sport are more restrictive when the symptoms are below the neck as they are thought to be more severe (16, 17). Unspecific symptoms, subclassified as “other” were headache and cough.

There were no significant differences with respect to the outcome parameters. [LVEF in MRI or echocardiography, occurrence of LGE, cardiac biomarkers, peak oxygen uptake (VO_2_peak)] between the patients with symptoms of an infectious disease in comparison to those without (see Table 2). The patients reporting of symptoms of an infectious disease were asked whether that led to a reduction in PA. The 13 patients (37.1%) who continued training showed a significantly higher LVEF in echocardiography (61.6 ± 7.5%) compared to those who stopped with their training (49 ± 15.7%). Even though, this was also true for the LVEF determined using MRI, this difference did not reach significance. All the other outcome parameters were comparable between these two groups (see Table 3).

**Table 2:** Mean values ± standard deviations of outcome parameters for participants who had experienced symptoms of an infectious disease two weeks prior to the onset of myocarditis and those who did not.

Outcome-Parameter	Infectious Disease around onset of myocarditis	No infectious disease around onset of myocarditis	Significance
CRP	26.7 ± 62.0	3.06 ± 7.0	0.169
(mg/L)			
Troponin	1,250.0 ± 2,698.0	1,138.3 ± 2,619.8	0.889
(ng/L)			
NT pro-BNP	2,405.1 ± 7,121.4	2,907.0 ± 5,221.0	0.805
(pg/ml)			
LVEF in echocardiography (%)	56.5 ± 13.3	51.3 ± 21.6	0.385
LVEF in MRI (%)	54.7 ± 8.8	56.6 ± 13.1	0.555
VO2peak	39.0 ± 10.5	39.6 ± 6.1	0.917
(ml/min/kg)			

MRI, cardiovascular magnetic resonance imaging; CRP, C-reactive protein; LVEF, left ventricular ejection fraction; NT-proBNP, N-terminal pro brain natriuretic peptide.

**Table 3:** Mean values ± standard deviations of outcome parameters for participants who kept training despite symptos of an infectious disease and those who did not.

Outcome-Parameter	No rest period despite infectious disease	Rest period as a consequence of infectious disease	Significance
CRP	6.9 ± 11.3	52.3 ± 106.3	0.214
(mg/L)			
Troponin	1,178.1 ± 1,800.2	949.1 ± 2,616.8	0.802
(ng/L)			
NT pro-BNP	158.4 ± 142.5	3,409.0 ± 8,518.5	0.331
(pg/ml)			
LVEF in echocardiography (%)	61.6 ± 7.5	44.5 ± 21.0	0.019
LVEF in MRI (%)	56.5 ± 8.3	54.9 ± 10.1	0.690
VO2peak	40.2 ± 13.8	37.5 ± 6.1	0.770
(ml/min/kg)			

MRI, cardiovascular magnetic resonance imaging; CRP, C-reactive protein; LVEF, left ventricular ejection fraction; NT-proBNP, N-terminal pro brain natriuretic peptide.

### 3.4. Recommendations

**Figure 3:**
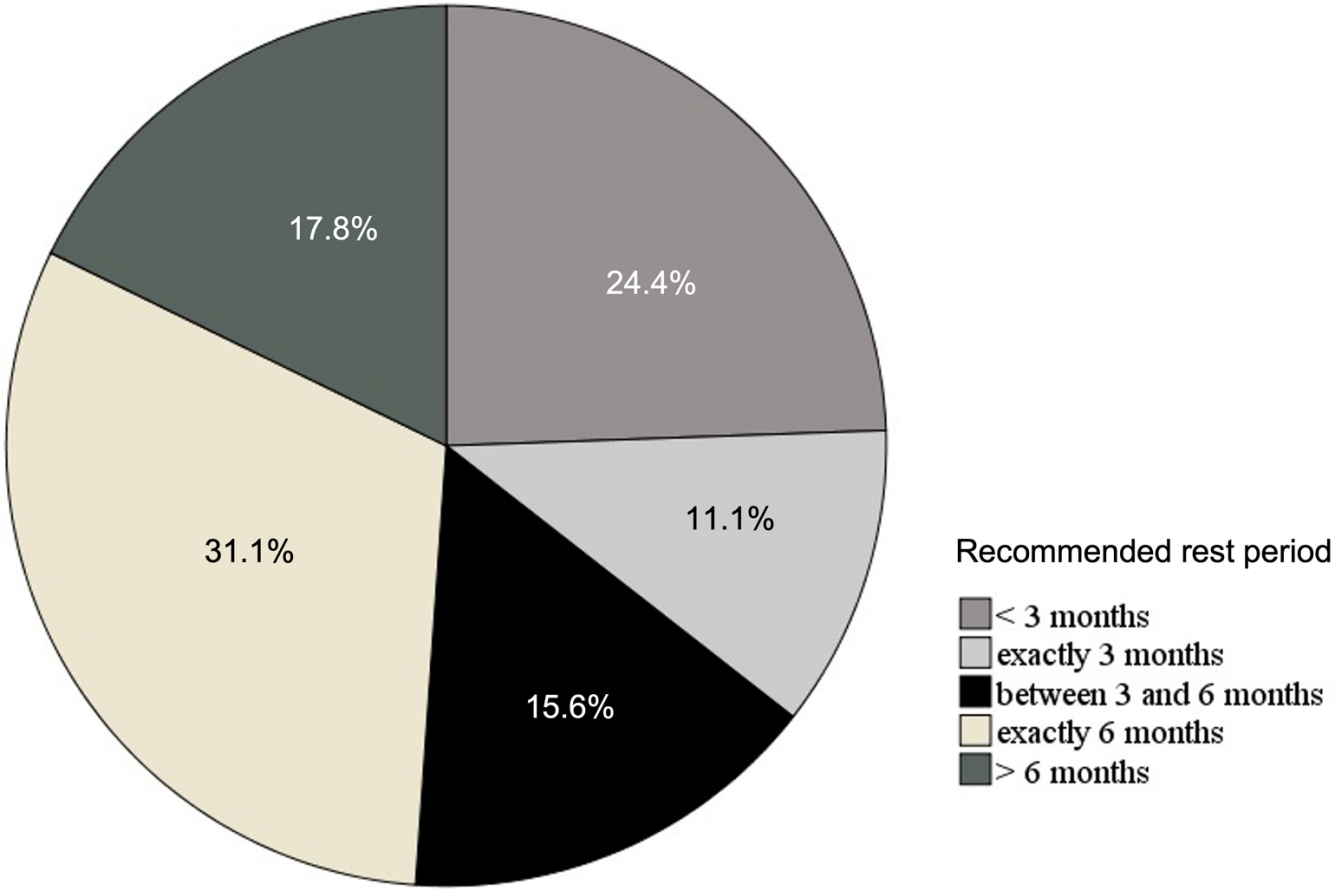
The rest periods recommended by the health care providers in %.

Most of the patients (82.8%) were recommended to stop physical activity after the onset of myocarditis by their medical care provider. However, the recommendations varied hugely with respect to return to sports from 4 weeks to 1 year (average 23.6 weeks). The recommended rest periods are represented in Figure 3. In only 44.8% of the cases the recommendations were in accordance with the recent recommendations from the European Society of Cardiology (ESC), whereas in more than half of the cases (55.2%) they were not (10). In those, where the recommendations were not adhered to, 57.9% of the cases were advised to return to sports after less than 3 months and in 42.1% the return to physical activity was longer than 6 months.

The criteria for return to sport include:

- normalization of EF and laboratory parameters (troponin, NT pro-BNP),
- no malignant arrhythmias in Holter Monitoring and/or during cardiopulmonary exercise test (CPET))

All necessary examinations needed for these criteria to be fulfilled were only performed in 15.5%. The most common examination performed before return to sports was the echocardiography with 70.7%. Less than half of the patients (48.3%) received cardiopulmonary exercise testing and Holter monitoring was conducted in 55.2%.

50 patients (86.2%) followed the recommendations with respect to the rest period. After return to sport 31 patients (53.4%) began with moderate intensity continuous training (MICT), 10 followed an individualized training protocol, 4 (6.9%) did not return to sports and 2 (3.4%) returned to competition level.

### 3.5. Return to sports

Only two patients (3.4%) experienced a relapse of their myocarditis of which one connected the relapse with physical activity. This 13-year-old male patient had been diagnosed with a Parvovirus B19 infection in a biopsy. Before the onset of myocarditis, he had participated in curricular physical activity and had played golf once a week for an hour. Even though his heart function was shown to be normal initially and during control examinations he only returned to sports over 12 months after the onset of myocarditis. A cardiopulmonary exercise test in combination with ECG before return to sports was normal without any signs of arrhythmias. Seventeen days after the last examination he experienced thoracic pain during long jump in school after also having had diarrhoea for three days. The ensuing MRI was suggestive of a relapse with new LGE in the basal and apical parts of the heart but no edema. A new biopsy revealed persistence of Parvovirus B19.

The other patient had a relapse unrelated to physical activity and is therefore not further discussed here.

The majority of patients had no symptoms when returning to sports (69%). Of those (31%) who had symptoms 7 reported of dyspnea, 7 of chest pain, and 9 of perceived tachycardia or arrhythmia.

Six months after the onset of myocarditis more patients did not participate in curricular activities than 6 months before the illness (22.4% vs. 10.3%). This high number of children not participating in school sports remained high after 12 months with 20.7%, even though they had had no severe cases of myocarditis with normal heart function (normal ejection fraction). However, these differences did not reach significance. Most of the subjects who had performed sports on a competition level (9 out of 12) returned to their previous competition sport. None of the competitive athletes experienced a relapse of the myocarditis. They also did not differ with respect to any of the outcome parameters from the group who did not participate in competition sports.

There was no significant difference between the type of extracurricular activity performed before the onset of myocarditis and 6 months later.

Regarding the transport to school or kindergarten nothing changed from before the onset of myocarditis and after. Before and after the onset of myocarditis 70% of all subjects chose the car or the bus to get to school.

## 4. Discussion

This study used data on a nationwide registry on myocarditis in children to investigate the relationship between myocarditis and sports.

As observed previously (14, 18, 19), there was a male predominance of patients with myocarditis who participated in this study which could be explained by protective effect of female hormones on the immune response (20). Male gender represents an independent risk factor for SCD (21, 22, 23). Most cases in our study ranged from 13 to 17 years of age. This is in accordance with previous reports in which two age peaks could be observed for myocarditis: <2 years of age and in adolescents (13–17 years) (14). Since we did not include children below the age of 6, we only observed the second age peak. As a consequence of the difficulty diagnosing myocarditis (24), this severe illness may be underdiagnosed especially in male adolescents. This in turn could be the cause for the high number of SCD due to myocarditis in this age group if not properly diagnosed, treated and provided with sufficient rest periods from sports.

In our study, the most popular sport disciplines were football and fitness which is in accordance with a previous study on sports related SCD in Germany (7). Since these are also the disciplines most commonly performed by the German youth this is probably merely a reflection of the number of children and adolescents performing these sports and not a reflection of the sports-specific risk with regards to myocarditis (25).

### 4.1. Around the onset of myocarditis

Interestingly, most of the patients did not reduce the amount of physical activity two weeks prior to the onset of myocarditis. However, most of the patients who experienced symptoms of an infectious disease (21 out of 35 patients) stopped training. From animal models it is known that intensive exertion leads to a higher amount of myocardial involvement (9, 26) and that even a short rest period (< 8 days) before returning to physical activity decreases the mortality due to myocarditis in mouse models (9). Recommendations about the return to sport after infectious disease (27) state that even simple infectious diseases require a sufficient period of convalescence with no sport. Still, 37% did not heed this advice and continued training as before, which did however not lead to a worse outcome.

The most common symptoms were respiratory symptoms as described previously (28), whereas abdominal symptoms were rare which is in contrast with findings from Japan (29). The reason for this discrepancy could lie in the fact, that our population was limited to children older than 6 years. Approximately a third of all patients experienced fever which led to a rest period from physical activity in all of them.

### 4.2. Recommendations

The fact that the recommendations for the return to sports varied widely between 4 weeks and 1 year reflects our clinical experience. The recommendations are based on a level of evidence of IIa at best (10). As myocarditis is a common cause of ventricular arrhythmias in its acute as well as its chronic phase (24, 30), a recommended time of rest period is paramount for ensuring the safety of the patients. In 19% of the cases the rest period was recommended to be less than 3 months, which is shorter than the current recommendations (10). On the other hand, 13.8% of the patients were advised to rest for more than 6 months, which is longer (10). Given the fact that sedentary behavior leads to serious long-term complications, especially in children and adolescents (31), such recommendations may seem protective but may cause more harm in the long run. The psychological consequences of prolonged breaks from physical activity notwithstanding, the consequences are especially harsh for athletes.

Only 15.5% of all patients received the recommended examinations (echocardiography, Holter Monitoring, CPET, laboratory examination, and ECG). Especially cardiopulmonary exercise testing and Holter monitoring are essential for unmasking malignant arrhythmias which are believed to be the main cause for SCD in athletes in the acute but also the chronic stages of myocarditis (30). Not performing this essential test before clearing children and adolescents for the return to sports could lead to missing malignant arrhythmias and thus represents a dangerous omission. Furthermore, performing an exercise test before clearing young people for the return to sport also enables the physician to state clear recommendations about taking up exercise again. In the case of a reduction in EF, heart rate values around the first ventilatory threshold can be provided for a gradual return to sports. In the case of a normal EF, the recommendation of returning to sports without restriction allows the child or adolescent to accept their recovery from myocarditis.

In our study most patients followed the advice from their health care providers. Especially athletes perceive an infection of the heart muscle as very concerning.

### 4.3. Return to sports

Only one patient reported of a sport-associated relapse of Parvovirus B19 myocarditis. The MRI was suggestive of a relapse and in the biopsy a persistence of Parvovirus B19 was revealed. However, ECG and cardiac biomarkers were normal, and the patient described no further symptoms after readmission. As Parvovirus B19 persistence has been observed even in asymptomatic individuals (32, 33) it is unclear whether the described symptoms were a true relapse.

Interestingly 31% of all patients described symptoms of dyspnoea, chest pain and perceived tachycardia when returning to sports but persisted with their physical activity without experiencing a relapse. All these symptoms could be interpreted as signs of a relapse or new onset of myocarditis or could stem from detraining. More information is needed on the specificity of symptoms regarding a possible relapse in order to provide the patients with clear recommendations.

There was an increase of children who did not participate in curricular physical activity 6- and 12-months after myocarditis. This is in part attributable to the recommendations from health-care providers. However, in part it is also a consequence of anxiety about the return to sports. After having been instructed to restrict physical activity for a recommended time frame, many parents and children develop anxiety about the return to sports. However, refraining from regular physical activity leads to a worsening of cardiorespiratory fitness (13). As little as an improvement of cardiorespiratory fitness (CRF) by 1 ml/kg/min can reduce the risk for developing overweight or obesity by 10% in 6 years (34). Improving CRF in childhood and adolescence is associated with a healthier cardiovascular profile (31). Given the fact that a physically active childhood enhances a physically active lifestyle over a life span (35), it is extremely important to keep young people active and exercising. It is therefore of the utmost importance to ensure that children and adolescents find their way back into physical activity after the recommended rest period.

### 4.4. Limitations

Out of 220 patients who were eligible to participate in this study, only 58 returned the questionnaire which in turn leads to a small study group. Not all questions were answered correctly. These incorrections consisted of some participants making two crosses where only one was possible or not answering the question at all. In these cases, no values were recorded for the given variable, limiting the number of answers. This further decreased the reliable data. Also, the study is retrospective in its approach so causalities could not be established.

### 4.5. Conclusion

Sports participation even on competition level before the onset of myocarditis was not associated with a more severe outcome in this retrospective study. There is still a discrepancy between current literature and actual recommendations given by health care providers with regards to the time of rest before return to sports. Before allowing a child or adolescent to start exercising again, normalization of EF and laboratory parameters (troponin, NT pro-BNP), and the exclusion of malignant arrhythmias in Holter Monitoring and/or during cardiopulmonary exercise test, are necessary. However, the necessary examinations for clearing a patient for return to sports are often not undertaken completely.

The level of evidence for establishing recommendation on physical activity after an episode of myocarditis needs to be increased and there is still a discrepancy between current literature and actual recommendations given by health care providers with regards to the time of rest before return to sports.

## Data Availability

If needed the dataset can be made available. Requests to access these datasets should be directed to isabelle.schoeffl@uk-erlangen.de.
